# Growth of microalgae and cyanobacteria consortium in a photobioreactor treating liquid anaerobic digestate from vegetable waste

**DOI:** 10.1038/s41598-023-50173-6

**Published:** 2023-12-19

**Authors:** Ewelina Sobolewska, Sebastian Borowski, Paulina Nowicka-Krawczyk, Tomasz Jurczak

**Affiliations:** 1 Interdisciplinary Doctoral School, Lodz University of Technology, 116 Żeromskiego street, 90-924 Lodz, Poland; 2 Department of Environmental Biotechnology, Faculty of Biotechnology and Food Sciences, Lodz University of Technology, 171/173 Wólczańska street, 90-530 Lodz, Poland; 3 Department of Algology and Mycology, Faculty of Biology and Environmental Protection, University of Lodz, 12/16 Banacha street, 90-237 Lodz, Poland; 4 UNESCO Chair On Ecohydrology and Applied Ecology, Faculty of Biology and Environmental Protection, University of Lodz, 12/16 Banacha street, 90-237 Lodz, Poland

**Keywords:** Chemical engineering, Environmental biotechnology

## Abstract

This research examines the biological treatment of undiluted vegetable waste digestate conducted in a bubble column photobioreactor. Initially, the bioreactor containing 3N-BBM medium was inoculated with *Microglena* sp., *Tetradesmus obliquus*, and *Desmodesmus subspicatus* mixture with a density of 1.0 × 10^4^ cells/mL and the consortium was cultivated for 30 days. Then, the bioreactor was semi-continuously fed with liquid digestate with hydraulic retention time (HRT) of 30 days, and the treatment process was continued for the next 15 weeks. The change in the microalgal and cyanobacterial species domination was measured in regular intervals using cell counting with droplet method on a microscope slide. At the end of the experiment, *Desmonostoc* sp. cyanobacteria (identified with 16S ribosomal RNA genetical analysis) as well as *Tetradesmus obliquus* green algae along with *Rhodanobacteraceae* and *Planococcaceae* bacteria (determined with V3–V4 16sRNA metagenomic studies) dominated the microbial community in the photobioreactor. The experiment demonstrated high treatment efficiency, since nitrogen and soluble COD were removed by 89.3 ± 0.5% and 91.2 ± 1.6%, respectively, whereas for phosphates, 72.8 ± 2.1% removal rate was achieved.

## Introduction

In the pursuit of sustainable development, many countries, including European Union members are increasingly turning to solutions such as Circular Economy (CE). Special attention is paid to the management of waste generated in various production processes^1–3^. Anaerobic digestion (AD) is a leading technology used for organic waste valorization and generation of energy. The anaerobic treatment process has many benefits, mainly production of methane-rich biogas resulting in reduction of greenhouse gas emissions^4^. At the same time, each biogas plant generates troublesome byproducts, mainly digestate. Raw digestate contains large amounts of organic solids, is rich in nutrients (nitrogen and phosphorus) but can also contain heavy metals and other toxic substances. The composition of digestate depends on many different factors, including feedstock composition, digester operating conditions or the source of microbial inoculum. Digestate can be processed in its original form or can be separated into liquid fraction (containing the majority of the volatile solids) and solid fraction (containing mostly insoluble particles). Solid digestate is often composted or processed into fertilizer, while liquid digestate is much more difficult to manage. Importantly, treated liquid digestate, can be re-used in biogas plants to dilute raw waste, which, in turn, could significantly reduce water consumption^5,6^. Unfortunately, conventional treatment methods applied to liquid digestate have many shortcomings, including high operating costs, generation of unwanted by-products, or weak nutrient removal^7^. Therefore, there is an urgent need to search for new, cost-effective, and environmentally friendly techniques, to treat liquid effluents generated in biogas plants.

Recently, increasing attention has been paid to the use of microalgae or microalgae-bacteria consortia systems. Microalgae are a diverse group of photoautotrophic organisms capable to bind CO_2_ and convert it into a valuable source of proteins, lipids and carbohydrates stored in biomass. Microalgae and cyanobacteria are able to tolerate and grow in a variety of environments, including liquid digestate. However, only a few types of microalgae (e.g., *Tetradesmus* sp., *Chlorella* sp., *Desmodesmus* sp.) have been reported to survive in such environment^8–10^. The presence of water and nutrients in the digestate allows for a cost-effective microalgae cultivation with simultaneous treatment^11^. It has been shown that microalgae–bacteria consortia are more suitable for digestate treatment than monocultures or microalgal axenic cultures. During photosynthesis, microalgae produce oxygen, further used by aerobic bacteria to mineralize organic compounds. Indigenous bacteria, on the other hand, provide microalgae with carbon dioxide, necessary for their growth. In addition, nitrifying bacteria can oxidize high concentrations of ammonium nitrogen that can be toxic to microalgae and thus improve the removal of nitrogen from the digestate. Through a symbiotic relationship, it is possible to obtain greater removal rates of pollutants and acquire highly valuable biomass^12–14^. To fully incorporate circular economy concept, a lipid-rich microalgal biomass cultivated on the liquid digestate can be further used as a sustainable feedstock for the 3rd and 4th generation biodiesel^7^. The dynamic relationship between bacteria and microalgae during the digestate treatment can be quite complex. It has been noticed that microalgae are able to compete with bacteria for nutrients, mainly in a phosphate-limiting environment^15^. Besides, it was found that the survival of pathogenic bacteria present in the digestate can be limited by various conditions of microalgae cultivation, such as high light intensity, variable pH, an increased concentration of dissolved oxygen or continuous stirring applied in the bioreactor^16^.

In the research reported by Sobolewska et al.^10^, a microbial consortium containing *Tetradesmus obliquus*, *Desmodesmus subspicatus* and *Microglena* sp. green algae was reported to efficiently remove both nutrients and organic pollutants from the liquid digestate in a small-scale laboratory installation. The following study summarizes the results of the undiluted liquid digestate treatment performed in a larger-scale photobioreactor operated under conditions closer to industrial implementation. Particular attention was paid to the microbial community and the roles of microalgae, bacteria and cyanobacteria in the treatment process. In contrast to the experiments described by other authors^12,17–19^, in this research, pretreatment of the substrate such as dilution or sterilization was not applied. Additionally, rarely used environmental isolates, including *Microglena* sp. (previously applied only in limited studies^10^) were employed allowing to achieve high removal rates of both nutrients and organic contaminants.

## Materials and methods

### Liquid digestate

The digestate was delivered from Warmia Fruit and Vegetable Processing Company Ltd. (Kwidzyn, Poland) as a product generated during anaerobic digestion of corn, peas and bean wastes. The liquid fraction was then obtained by centrifuging the digestate for 10 min at 4000 rpm (4800 G) using a Rotina 420 centrifuge (Hettich, Switzerland). The substrate was not subjected to the initial pre-treatment such as sterilization or dilution as such processes are usually omitted on a technological scale^20^. During the experiment, the liquid digestate was stored at 4 °C in closed plastic containers. Before each batch cycle, the substrate was first brought to room temperature. To confirm substrate stability, a full psychochemical analysis of the liquid digestate was performed 4 times throughout the study period according to the methodology described in “Chemical and physical analyses”. The characteristic of the liquid digestate is presented in Table 1.

**Table 1:** Chemical and physical composition of liquid digestate*

Indicator	Mean value ± standard deviation
pH	7.75 ± 0.13
Total solids (g/kg)	3.10 ± 0.02
Volatile solids (g/kg)	1.59 ± 0.03
Volatile solids (%TS)	51.36 ± 0.77
Total suspended solids (mg/L)	785.55 ± 118.10
Turbidity (FAU)	472.52 ± 99.14
Apparent color (units)	12,078.10 ± 2079.80
True color (units)	10,998.13 ± 2015.33
Total chemical oxygen demand (mgO_2_/L)	7990.00 ± 540.00
Soluble chemical oxygen demand (mgO_2_/L)	7890.00 ± 485.00
Total volatile fatty acids (mg/L)	3183.42 ± 989.67
Ammonium nitrogen (mg/L)	581.50 ± 81.50
Nitrates (mg/L)	1020.16 ± 103.70
Nitrites (mg/L)	10.21 ± 2.71
Total nitrogen (mgN/L)**	685.74 ± 87.29
Orthophosphates (mg/L)	350.27 ± 20.11
Zinc (mg/L)	15.04 ± 1.62
Aluminum (mg/L)	10.35 ± 8.58
Copper (mg/L)	5.70 ± 0.43
Iron (mg/L)	4.07 ± 2.12
Chlorides (mg/L)	217.19 ± 30.11
Sulfides (μg/L)	1553.00 ± 607.08
Sulfates (mg/L)	0.00 ± 0.00

*Mean values ± SD from 12 measurements.

**Calculated as the sum of all nitrogen forms (NH_4_^+^, NO_3_^−^, NO_2_^−^) computed from Eq. (2).

### Inoculum

Bioreactor was inoculated with *Microglena* sp., *Tetradesmus obliquus*, and *Desmodesmus subspicatus* microalgae mixture of 1.0 × 10^4^ cells/mL cell density. The respective species were isolated, genetically identified and their sequences were deposited in the National Center for Biotechnology Information (NCBI) for the *rbcl* gene under accession number of ON457158–ON457161, and ON426490–ON42693 for ITS region (www.ncbi.nlm.nih.gov, accessed on 20 April 2022), which was described by Sobolewska et al.^10^. For the initial microalgae multiplication, a cultivation on Bold Basal Medium with threefold nitrogen concentration (3N-BBM), prepared in accordance with Andersen^21^ was used.

### Photobioreactor design and operation

**Figure 1:**
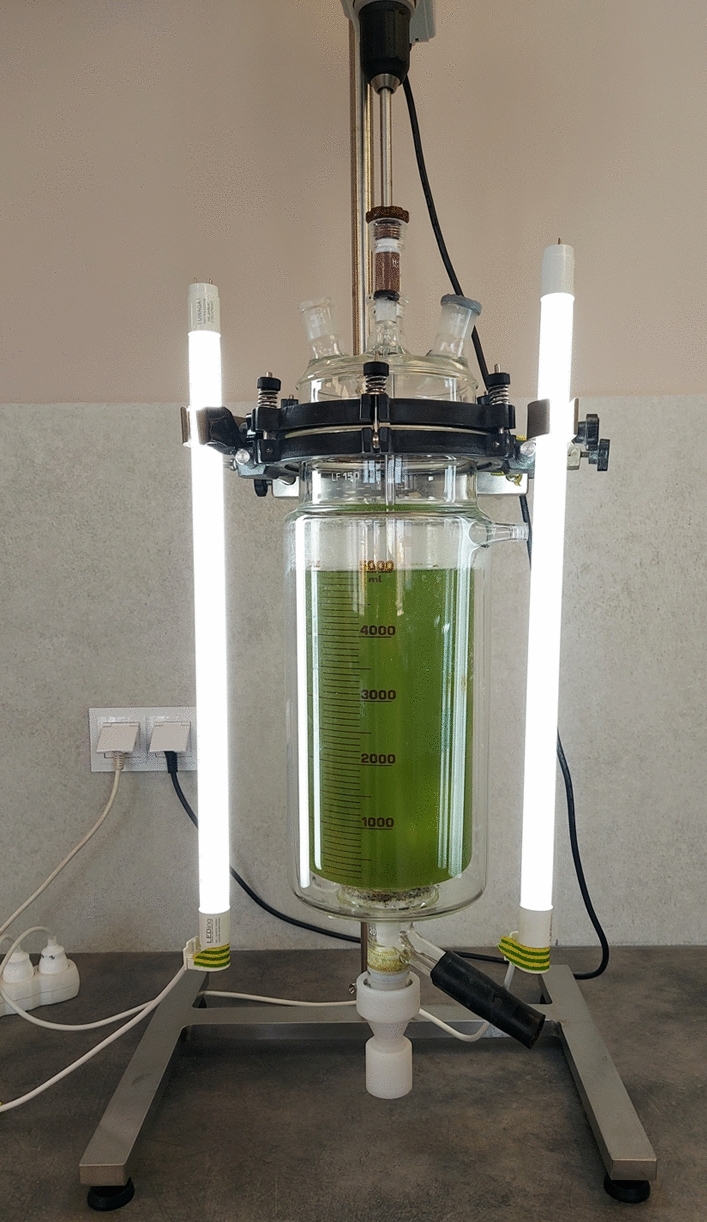
Installation with a photobioreactor used for the experiment.

The experiment was carried out semi-continuously in a glass photobioreactor with a 5 L working volume and continuous stirring at 150 rpm (Fig. 1). The reactor was illuminated with LED lamps in the 14 h light/10 h dark cycle mode. T8 LED Linio model lamps with the following parameters: 1440 lm (luminous power), 14.5 W (electric power) and 6000 K (color temperature) were employed for the process. The illumination intensity was set at 3500 Lux. A membrane pump (Yasunaga, Japan) delivered air to the reactor at a flow rate of 0.2 L/L/min. Initially, the bioreactor was filled with a 3N-BBM medium, inoculated with a 10% v/v microalgae consortium, and operated in a batch mode for 30 days to increase the biomass concentration. After this period, the reactor was semi-continuously fed with the liquid fraction of the digestate for the next 15 weeks. Hydraulic retention time (HRT) was set to 30 days following the previous experiences^10,22^. Once a day, 167 mL of the liquid was withdrawn from the reactor and the same volume of fresh liquid digestate was introduced. As the biomass was not recycled back to the bioreactor, the hydraulic retention time was equal to solid retention time (SRT). Feeding and discharge operation was carried out by a peristaltic pump. Once a week total chemical oxygen demand (tCOD), total suspended solids (TSS), turbidity as well as biomass parameters, including optical density (OD), the concentration of chlorophyll *a* (chl-*a*) and the microalgal cell abundance were measured in the effluent. pH, soluble COD (sCOD), ammonium nitrogen, nitrates, nitrites, orthophosphates were determined in the effluent after filtration. The efficiency of nutrient and organic contaminant removal was calculated for steady-state period with Eq. (1). Steady-state period was defined as an experimental period during which most of the determined indicators were not changing by more than 10%. The removal efficiency of individual indicators was calculated assuming theoretical, complete separation of microalgal biomass.

1 $$ {\text{R}}\left[ \% \right] = \left( {{\text{c}}_{{1}} {-}{\text{c}}_{{2}} } \right) \times {1}00/{\text{c}}_{{1}} $$

Where R—removal efficiency of an indicator (PO_4_^3−^; NH_4_^+^, NO_3_^−^, NO_2_^−^ (total nitrogen); COD); c_1_—concentration of an indicator in the fresh liquid digestate; c_2_—concentration of an indicator in the effluent.

### Analytical methods

#### Chemical and physical analyses

All chemical analyses were performed using an UV–VIS DR6000 spectrophotometer (Hach Lange, Loveland, CO, USA) and Hach Lange tests. The concentrations of chemical oxygen demand, total volatile fatty acids, nitrates, nitrites, ammonium nitrogen and orthophosphates were measured by LCK214, LCK365, NitraVer 5, NitriVer 3 tests, a modified Nessler method (no. 8038) and a PhosVer 3 test (no. 8048), respectively. The contents of nitrogen in ammonium nitrogen, nitrates and nitrites were then summed up to calculate the total nitrogen (Eq. 2). Measurements of organic nitrogen forms were not performed considering the fact that liquid digestate primarily consists of ammonium nitrogen and only low amounts of organic nitrogen forms^23,24^. The concentration of chlorides, sulfides and sulfates were measured by a thiocyanate method no. 8113, a methylene blue method no. 8131 and a SulfaVer 4 method no. 8051, respectively. In addition, an AluVer method no. 8012 (for aluminum), CuVer 1 method no. 8506 (for cooper), FerroVer method no. 8008 (for iron), Zincon method no. 8009 (for zinc) were applied. The Hach Lange kits were applied to diluted samples to match detection ranges of the tests used. Final indicator values were calculated including the dilution factor used. The color, turbidity and total suspended solids (TSS) were assessed in accordance with 8025, EN ISO 7027 and 8006 methods, respectively. Total solids (TS), volatile solids (VS) and pH were examined in accordance with Standard Methods^25^. Biomass productivity was calculated in accordance to methodology described in Min et al.^26^ based on TSS values:

2 $$ {\text{Total nitrogen }}\left[ {{\text{mgN}}/{\text{L}}} \right] \, = \, 0.{778} \times {\text{ cNH}}_{{4}}^{ + } + \, 0.{226 } \times {\text{ cNO}}_{{3}}^{ - } + \, 0.{3}0{4 } \times {\text{ cNO}}_{{2}}^{ - } $$

Where 0.778—nitrogen molar mass to NH_4_^+^ molar mass ratio; 0.226—nitrogen molar mass to NO_3_^−^ molar mass ratio; 0.304—nitrogen molar mass to NO_2_^−^ molar mass ratio; c [mg/L]—concentration of a respective nitrogen form (NH_4_^+^/NO_3_^−^/NO_2_^−^).

#### Biomass analysis

##### Optical density

Optical density was measured at 680 nm, which correlated with the second peak of chlorophyll absorption, and reflected the concentration of biomass in the cell suspension^27^. This value was also confirmed experimentally by scanning microalgal culture in the absorption spectrum between 540 and 900 nm. The analysis was performed using a UV–VIS DR6000 spectrophotometer (Hach Lange, Loveland, CO, USA).

##### Quantitative and qualitative microalgae–cyanobacteria structure in photobioreactor

The density of microalgal and cyanobacterial cells in the bioreactor was established using a droplet method on a microscope slide. Only healthy, active cells were counted. The abundance of cells was calculated based on Eq. (3). Additionally, the percentage share of the individual taxa in examined biomass was ascertained.

3 $$\mathrm{Dx }[{\text{cells}}/{\text{mL}}] = (\mathrm{zi }\times \mathrm{ Ap})/(\mathrm{Ai }\times \mathrm{ V})$$

where Dx—the density of cells in 1 mL of culture, zi—cell number of particular taxa, Ap—the cover-slip surface area (= 400 mm^2^), Ai—the surface area observed under the microscope (mm^2^), V—the sample volume placed on a microscope slide (cm^2^).

##### Chlorophyll a concentration

To determine the chlorophyll *a* concentration, the procedure according to Lee et al.^28^ was used. After extraction performed with a 90% methanol solution, the absorbance was measured spectrophotometrically at the wavelengths of 665 nm and 650 nm. For these measurements, a 10 mm path length of the cuvette was applied. The chlorophyll *a* concentration was calculated based on Eq. (4):

4 $$ {\text{chl - }}a\left[ {{\text{mg}}/{\text{L}}} \right] = \left( {{16}.{5} \times {\text{A}}_{{{665}}} } \right) - \left( {{8}.{3} \times {\text{A}}_{{{65}0}} } \right) $$

#### Taxonomic analysis of cyanobacteria

Cultivation of *Desmonostoc* has been done to multiply its biomass in the form of unialgal culture, prior to isolation of its DNA, to obtain high DNA yields of good quality. Isolation of cells and multiplication of strain biomass for DNA isolation protocols is a standard procedure, widely used in algal molecular characterization. The cyanobacteria taxa from bioreactor were identified using an integrated morphological and molecular approach. Individual trichomes were isolated using microcapillary and inverted light microscope (Nikon Eclipse Ti2-E, Precoptic Co., Warsaw, Poland) via passaging through a series of BG-11 drops to washed out excess of bacteria and transferred into sterile 1.5% BG-11 agar plates. Biological material was cultivated under artificial light from fluorescent tubes (Osram FLUORA T8 L 36W/77) of 2800 Lux in a 16 h/8 h day/night period, with a temperature of 22  ± 0.2 °C and air humidity of 50 ± 5%. The genomic DNA was isolated using DNeasy PowerBiofilm Kit (Qiagen, Hilden, Germany). The 16S rRNA molecular region was amplified by PCR reaction, using primers 359F^29^ and 23S30R^30^ with Color Perpetual Taq PCR Master Mix (Eurx, Gdansk, Poland) in 50 μL reaction volume under the reaction conditions as in Nowicka-Krawczyk et al.^31^. The results of PCR were visualized on 1.5% agarose gel and the sample was purified using QIAquick PCR Purification Kit (Qiagen, Hilden, Germany). The sample was sequenced commercially using Sanger Sequencing and the primers of PCR reaction. The sequences were assembled and clean up in Geneious software ver.11.0.15 and screened using BLAST in nucleotide collection of NCBI GenBank database. Obtained sequence was submitted to GenBank under the OQ792152 accession number.

#### Determination of microcystins

To confirm whether *Desmonostoc* sp. cyanobacteria, present in the tested culture do not produce cyanotoxins and can only positively contribute to nitrogen and phosphorous removal, a HPLC-DAD analysis was conducted for the presence of microcystin-LR (MC-LR) and microcystin-RR (MC-RR). To analyze the microcystin (MC) content in freshwater material, the 13 mL of sample was filtered through GF/C Whatmann filter. The material collected on the filter was extracted in a 75% solution of aqueous methanol and then the sample was sonicated for 30 s in the Misonix (Farmingdale, NY, USA) ultrasonicator with a ultrasonic liquid processor XL. The extract was then centrifuged twice at 11,000×*g* for 10 min at 4 °C in the Eppendorf 5804 centrifuge (Germany). The supernatant was collected and together with a water subsample after filtration process were evaporated to dryness in the SC110A Speedvac^®^ Plus, ThermoSavant (Holbrook, NY, USA). The samples were redissolved in 500 μL of 75% aqueous methanol and filtered through a Gelman GHP Acrodisc 13 mm syringe filter with a 0.45 μm GHP membrane and minispike outlet (East Hills, NY, USA). A chromatographic separation was performed using an Agilent (Waldbronn, Germany) 1100 series HPLC system consisting of a degasser, a quaternary pump, a column compartment thermostat set at 40 °C, and a diode array detector operated at 200–300 nm on a Merck (Darmstadt, Germany) Purospher STAR RP-18e column (250 mm × 4 mm I.D. with 5 μm particles) protected by a 4 mm × 4 mm guard column. The mobile phase consisted of water (solvent A) and acetonitrile (solvent B), both containing 0.05% trifluoroacetic acid. The flow rate was 1 mL/min with the following linear gradient program: 25% B at 0 min, 70% B at 5 min, 70% B at 6 min, 25% B at 6.10 min; stop time, 9 min. The injection volume was 20 μL. The content of microcystin-LR (MC-LR) and microcystin-RR (MC-RR) in the samples was analyzed by comparing the retention time and UV spectrum (200–300 nm with an absorption maximum at 238 nm). For calibration of the HPLC analysis, commercial standards of MCs (EnzoLife Sci.) were used.

#### Metagenomic analysis

At the end of the experimental run, the biomass from the reactor was analyzed metagenomically (V3-V4 of 16S rRNA) for bacterial community. The collected biomass was stored at − 20 °C until analysis. For the isolation of genomic DNA, Genomic Mini AX Bacteria + kit (A&A Biotechnology, Poland) was applied. The FastPrep-24 homogenizer and zirconia beads were used to perform mechanical cell lysis. The PCR was carried out applying Q5 Hot Start High-Fidelity 2X Master Mix (New England Biolabs, United Kingdom). Dedicated primer sequences 314F (TCGTCGGCAGCGTCAGATGTGTATAAGAGACAGCCTACGGGNGGCWGCAG) and 785R (GTCTCGTGGGCTCGGAGATGTGTATAAGAGACAGGACTACHVGGGTATCTAATCC) were used to amplify the selected region. Amplification, indexing as well as library quantification were executed on an Illumina MiSeq system in accordance to the procedure of "16S Metagenomic Sequencing Library Preparation" with Q5 High-Fidelity Polymerase applied as the only modification method. Next Generation Sequencing (NGS) was conducted on a MiSeq apparatus, in 2 × 300 paired-ends, using the Illumina v3 kit (Illumina, USA). Bioinformatic analysis ensuring the classification of reads to the species level was performed with the QIIME 2 software package based on Silva 138 (reference sequence database). Metagenomic data stored in fastq format has been deposited to the NCBI database. The Sequence Read Archive (SRA) tool was used to create a bioproject with accession number of PRJNA922144, and generated BioSampleAcc. number of SAMN32643820. The 16S rRNA gene fragments were matched to the appropriate taxonomical levels (from phylum to species) in order to determine phylogenetic microbial diversity.

### Statistical analysis

The measurements were conducted in triplicates and results were expressed as mean value ± standard deviation. Mean values, standard deviation, error bars and the analysis of correlation were determined in Microsoft Excel 365 (version 2211).

## Results and discussion

### Characteristic of liquid digestate

The composition of liquid digestate is depicted in Table 1. The liquid was characterized by alkaline pH, typical for plant waste digestate^32^, and COD greater than that typically reported in the literature^33^. It also contained a significant concentration of nitrates (1020 mg/L) and orthophosphates (350 mg/L). The analysis for metals showed the highest concentration of zinc (15.04 mg/L) and lowest content of iron (4.07 mg/L). The digestate was also characterized by relatively low salinity with 217 mg/L of chlorides and 1.5 mg/L of sulfides.

### Photobioreactor performance

**Figure 2:**
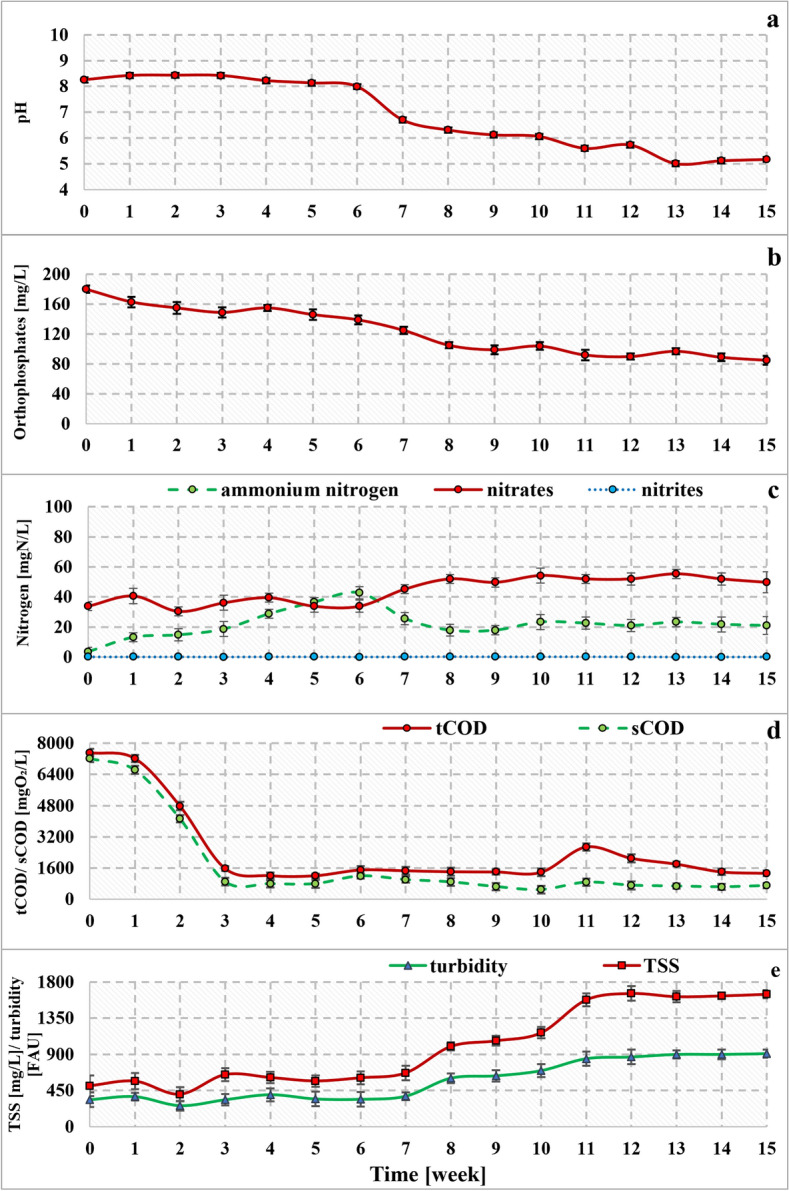
Changes in pH (**a**), orthophosphates (**b**), nitrogen (**c**), COD (**d**), turbidity and TSS (**e**) in the photobioreactor effluent.

The liquid digestate treatment process was evaluated based on the removal rates of nutrients and organic carbon (expressed in COD). Changes in pH, nitrogen, orthophosphates, total and soluble COD are shown in Fig. 2. Characteristics of effluent collected from the bioreactor and pollutant removal rates are shown in Table 2.

**Table 2:** Characteristics of the effluent from the photobioreactor and the pollutants removal efficiency in the steady state period.

Indicator	Mean value ± standard deviation	
	Concentration	Removal rate (%)
Ammonium nitrogen	27.1 ± 2.8 mg/L	X
Nitrates	230.6 ± 8.6 mg/L	X
Nitrites	0.7 ± 0.1 mg/L	X
Total nitrogen*	73.4 ± 3.8 mgN/L	89.3 ± 0.5
Orthophosphates	95.1 ± 7.3 mg/L	72.8 ± 2.1
Soluble COD	696.9 ± 128.0 mgO_2_/L	91.2 ± 1.6
Total COD	1687.5 ± 482.9 mgO_2_/L	78.9 ± 6.0

X—removal rate not determined for individual nitrogen compounds due to the fact that nitrates and nitrites are formed during the oxidation of ammonium nitrogen.

*Calculated as the sum of all nitrogen forms (NH_4_^+^, NO_3_^−^, NO_2_^−^) computed from Eq. (2).

#### Removal of phosphates

Phosphorus plays a significant role in the growth and energy metabolism of microalgae. Microalgae mainly use inorganic phosphates and transform them into organic compounds by the process of phosphorylation^34^. In the 3N-BBM medium used for initial cultivation of microalgae, phosphorous was supplied with K_2_HPO_4_ and KH_2_PO_4_, salts known as one of the most bioavailable sources of this nutrient for microalgae^35^. Regarding the liquid digestate used in the study, the phosphorous concentration (calculated based on orthophosphates) remained below the threshold value of 0.179 g/L reported in the literature to be inhibitory for microalgae^36^. It can be hypothesized that an increased removal rate of phosphate could be due to a generally high initial concentration of this nutrient. This phenomenon is known as “luxury phosphorous uptake”, which relies on greater phosphate accumulation by microorganisms cultivated under stressful conditions as described previously^37,38^. During the experimental run, the pH value in the effluent remained at approximately 8 for the first 6 weeks. After this period, a decrease in pH was observed, which corresponded with a drop in orthophosphates. This further indicates that removal of phosphates resulted from microbial uptake rather than precipitation^34^. The most noticeable phosphate decrease strongly corresponded with the significant growth of microalgae reported between the 5th and 11th week. In the same time, an intensive development of cyanobacteria (and probably bacteria) was also observed. It should be noted that these microorganisms usually exhibit higher ability to fix phosphorous than microalgae^39,40^. After reaching steady state (8–15 weeks), the average concentration of orthophosphates dropped to 95.1 ± 7.3 mg/L (Fig. 2b) and the calculated removal rate for this period was 72.8 ± 2.1%. The removal rate of phosphates was therefore greater than that reported in the previous experiments described by Sobolewska et al.^10^. This could have been influenced by the appearance of cyanobacteria *Desmonostoc* sp., which could enhance the treatment process (further discussed in “Biomass parameters monitoring” and “Relative abundance of microbial community in bioreactor”). Higher removal rates of 78.3 ± 1.1% were reported by Sayedin et al.^41^ describing the treatment of thin stillage anaerobic digestate by microalgae. However, the cited authors investigated diluted liquid digestate after preliminary struvite recovery. In another study, the liquid digestate from maize silage and distillery stillage anaerobic processing was subjected to biological treatment using two microalgal species^42^. The authors used centrifugation/distillation and dilution as pretreatments and achieved 94.2% and 70.4–87.4% phosphorous removal rates for *Arthrospira platensis* and *Chlorella vulgaris,* respectively. In contrast, Deng et al.^43^ treated sterilized anaerobic digestate from swine manure reaching 20.0–29.7% of total phosphorous removal.

#### Nitrogen removal

Nitrogen is a nutrient necessary for the growth and metabolic processes of all living organisms. Eukaryotic microalgae assimilate inorganic nitrogen in the form of ammonium, nitrates and nitrites while cyanobacteria can also transform atmospheric nitrogen^34,44^. Although nitrates are a preferred nitrogen source, ammonium nitrogen is usually assimilated by microalgae faster^45,46^. The ability to assimilate various nitrogen forms or their inhibitory concentrations are species and strain dependent^47^. In the 3N-BBM medium, ammonium nitrogen was not present (NaNO_3_ used as a primary nitrogen source), while in digestate both ammonium nitrogen and nitrates were. An increased ammonium concentration could potentially inhibit the microalgal growth, however, in the previous study reported by Sobolewska et al.^10^, the *Microglena* sp., *Tetradesmus obliquus*, and *Desmodesmus subspicatus* consortium could successfully grow in the same substrate. The concentration of nitrates showed a slightly increasing trend throughout the entire run, whereas ammonium nitrogen increased up to 6th week, and then dropped. From the 8th week of the experimental run, both nitrogen forms remained at relative constant levels suggesting stabilization of the process (steady-state operation). After the 6th week of the experiment, a simultaneous decrease in both pH and ammonium nitrogen concentration was reported (Fig. 2a,c) suggesting the possible occurrence of nitrification. The presence of nitrifying bacteria within the reactor community was also confirmed in the metagenomic analysis. However, as dissolved oxygen and/or CO_2_/carbonates were not measured, it cannot be unambiguously confirmed that nitrification was the main process, which contributed to nitrogen removal. Apart from nitrification, ammonium nitrogen could have been removed through its microbial assimilation by bacteria and microalgae as well as cyanobacteria. The activity of nitrifying bacteria contributes to the reduction of free ammonia concentration, which might be toxic to microalgae. Hence, the coexistence of microalgae and nitrifying bacteria could increase the removal rate of nitrogen and overall bioreactor performance as reported previously^14,48^. Considering the steady-state operation, nitrogen was removed in 89.3 ± 0.5%, and its average concentration in the effluent was 73.4 ± 3.8 mgN/L. Lower treatment efficiency was reported by Xu et al.^49^ for piggery anaerobic liquid digestate. It should be noted that authors used initial substrate sterilization and dilution achieving total nitrogen removal rates of 58.39–74.63%. Pizzera et al.^17^ investigated diluted liquid agro-digestate treated by *Chlorellaceae* and *Scenedesmaceae* reaching 20 ± 29% total nitrogen removal rates. In contrast, Khalid et al.^50^ achieved 96% NH_4_^+^ removal rates for recycled, sterilized agricultural wastewater. A similar treatment efficiency was also demonstrated by Sayedin et al.^41^.

#### COD and TSS changes

Carbon, along with nitrogen and phosphorus, is an important component used by microalgae. It can be consumed either directly in the form of soluble carbonates, or converted into carbon dioxide and then utilized through photosynthesis^34^. Although some microalgae can directly assimilate organic carbon^51^, the removal of organic substances is mainly due to bacterial activity in mixed cultures. On the other hand, microalgae can provide oxygen necessary for decomposition of organic substances by bacteria, which, in turn, release carbon dioxide used by photosynthetic organisms^44,52^. The observed COD reduction was therefore associated with the activity of both co-existing microbial groups. As shown in Fig. 2d, a sharp decrease in total and soluble COD was observed after the first week of the treatment process. From the third week, the values of total and soluble COD remained at a constant low level of 1687.5 ± 482.9 mgO_2_/L and 696.9 ± 128.0 mgO_2_/L, respectively. For that period, the calculated sCOD removal rate exceeded 90% while the corresponding value for tCOD was 79%. Zhu et al.^53^ noted lower efficiency for non-sterilized dairy liquid digestate (DLD) but the authors employed axenic cultures of *Chlorella vulgaris.* They observed that with increasing DLD load, the sCOD removal rate also increased from 1.5% (25% DLD) to 32.2% (100% DLD). The sCOD removal rate of 29 ± 17% was reached by Pizzera et al.^17^, who investigated diluted liquid digestate from agricultural waste processing. Likewise, Wang et al.^54^ treated diluted dairy manure digestate using wild-type microalgae. Depending on the dilution used, the sCOD removal rates of 27.4–38.4% were achieved. Bankston and Higgins^18^ showed that axenic cultures of *Chlorella sorokiniana* and *Auxenochlorella protothecoides* cultivated on diluted, sterilized digestate may increase soluble COD whereas the presence of anaerobic digestion microorganisms suppress this effect to a small extent.

**Figure 3:**
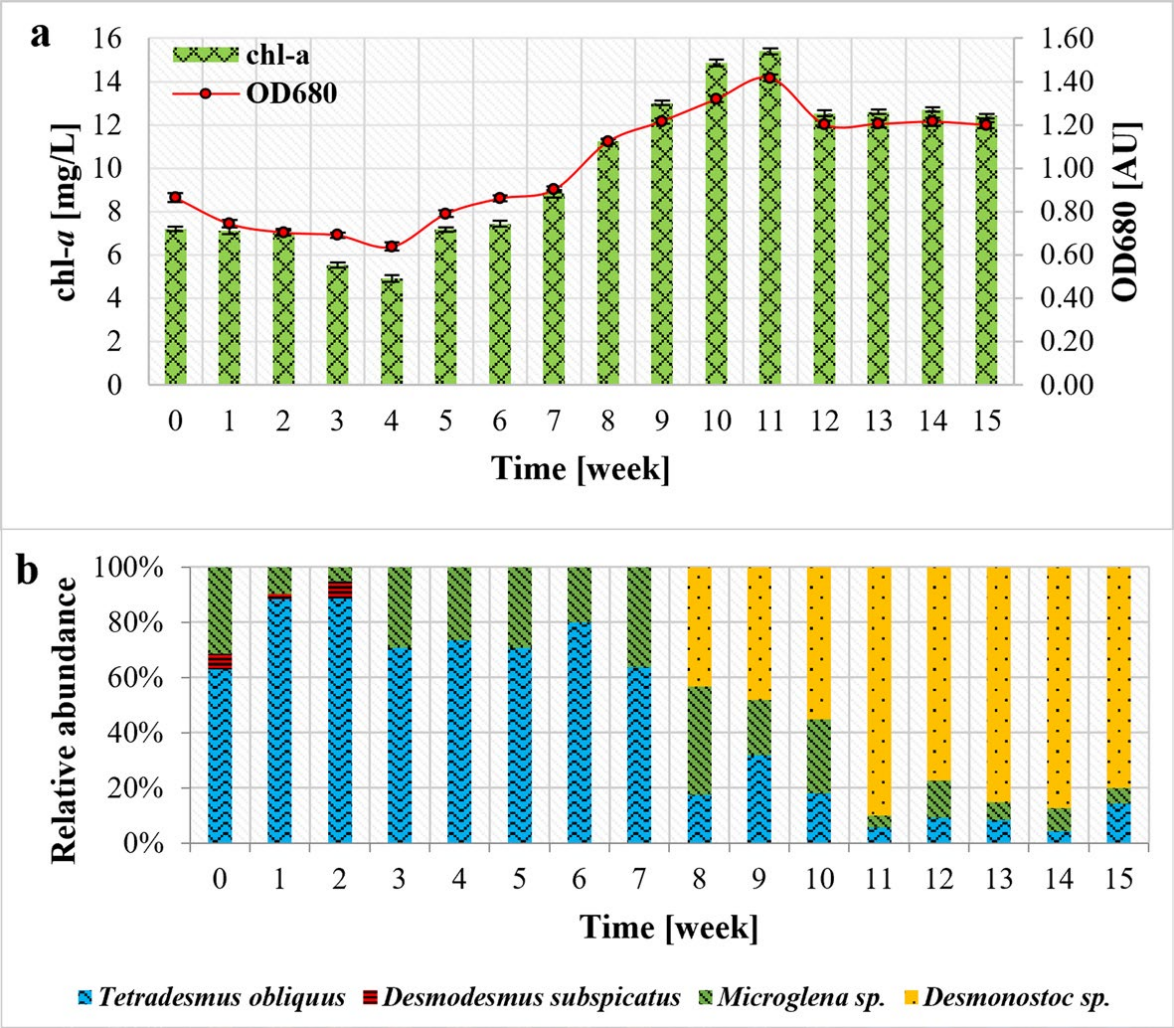
Changes in optical density, chlorophyll *a* concentration (**a**) and percentage share of particular taxa of microalgae and cyanobacteria (**b**).

In contrast to COD, turbidity and especially TSS showed an increasing trend throughout the experiment (Fig. 2e). Turbidity is directly influenced by light dispersion resulting from the presence of colloids, suspended particles, pollutants and microorganisms^55^. This may also explain the correlation between turbidity, optical density and the abundance of microalgae (which form colloidal structures) as illustrated in Fig. 3. Although all microorganisms can influenced turbidity and TSS, it seems that cyanobacteria and microalgae were mainly responsible for this since a correlation between chlorophyll *a* concentration (Fig. 3a), TSS and turbidity was noticed. Biomass productivity, calculated as a function of TSS (see “Chemical and physical analyses”) was recorded at 11 mg/L/day. Given the proper separation of treated effluent, the remaining biomass can be valorized to generate biofuels or other value-added products (pigments, nutraceuticals, fertilizers)^56,57^.

### Biomass parameters monitoring

During the experimental run, the growth of microalgal biomass was monitored with optical density, chlorophyll *a* concentration and cell count determinations (Fig. 3). After initial cultivation, for the first 7 weeks *Tetradesmus obliquus* followed by *Microglena* sp. were the most abundant microalgae. Then, the number of *T. obliquus* cells gradually decreased, which corresponded to a pH drop below 7. The growth of *T. obliquus* is strongly dependent on environmental pH with the optimal value close to 7. Lower pH may result in cell division inhibition^21,58^. Simultaneously, from the 8th week, the growth of heterocytous bacteria was also observed. The morphological features of trichomes allowed to predict the *Nostoc* sensu lato group. However, molecular analysis (BLASTn) confirmed the presence of *Desmonostoc* species with 100% coverage and 100% identity to *Desmonostoc* sp. PCC7422 (HG004586). As the digestate used in this study had not been subjected to pre-treatment processes (see “Liquid digestate”), it contained various native microorganisms. It can therefore be assumed that singular *Desmonostoc* sp. cells were introduced to the bioreactor along with the digestate, and their rapid multiplication was accelerated by the temperature increase and pH drop^59^. For example, *Desmonostoc muscorum* was observed in soils at pH below 5.0^60^. In general, representatives of this genus prefer slightly acidic to neutral environment (pH 5.3 up to 7.0)^61^. A rapid growth of cyanobacteria was reflected by an increase in optical density, chlorophyll *a* concentration, as well as TSS and turbidity. A strong correlation between these indicators (R > 0.85) was found. Although it is commonly believed that cyanobacteria can produce toxins hazardous for other organisms, their release is usually strain dependent^62^. Cyanotoxins can be released during water treatment processes as a result of mechanical and chemical stress factors^63^. For instance, the use of aluminum sulfate or chlorine dioxide for water and wastewater treatment can contribute to the lysis of cyanobacteria cells and caused the release of microcystins into water as demonstrated for full-scale applications^64,65^. However, a HPLC–DAD analysis (described in “Determination of microcystins”) confirmed the absence of MC-LR and MC-RR microcystins in the analyzed samples. Moreover, the presence of blue-green algae can also facilitate bioremediation and biomass generation, which can be further processed to value added products^66^.

### Relative abundance of microbial community in bioreactor

**Figure 4:**
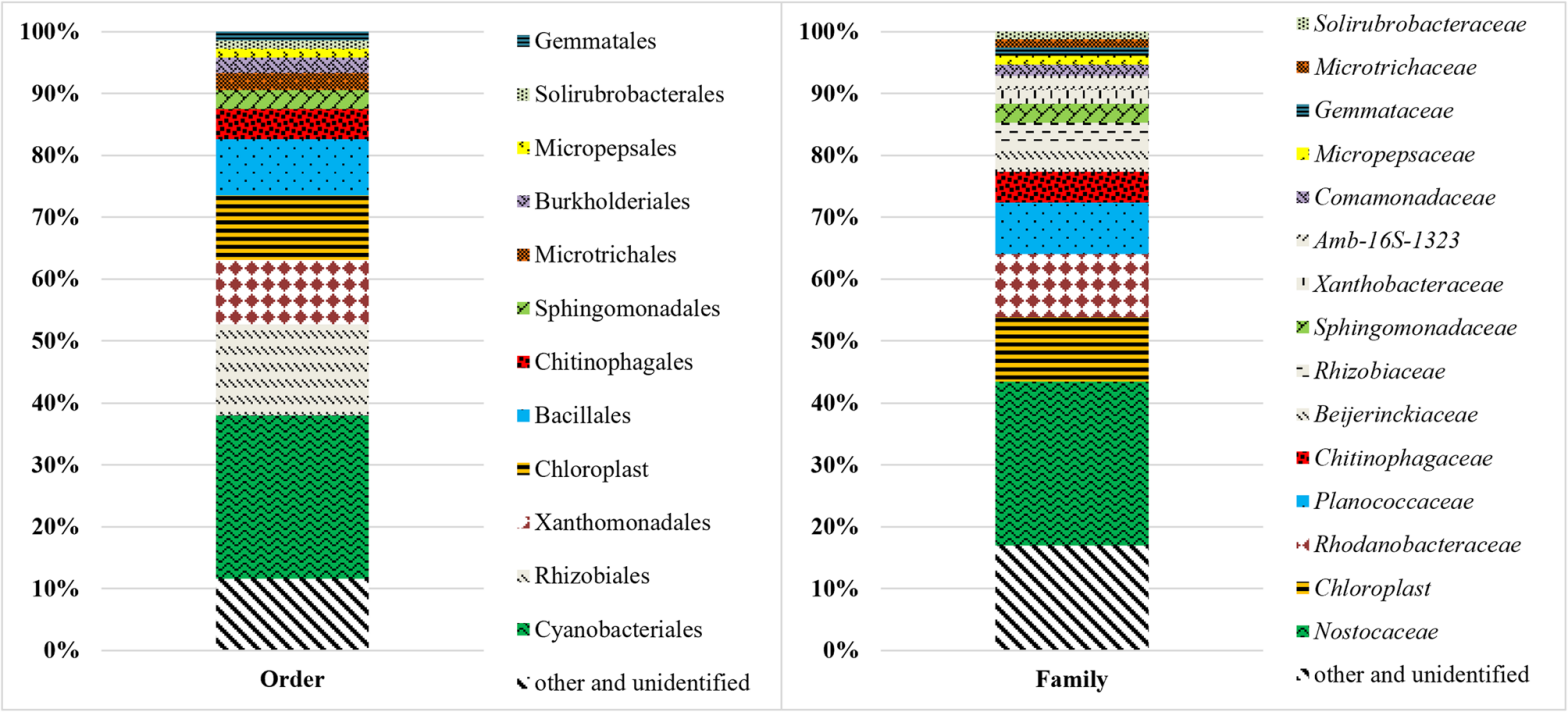
Microbial community of the reactor biomass at an order and family level.

Metagenomic analysis of the biomass collected at the end of the experimental run allowed to assess the microbial community (bacteria and archaea) present in the photobioreactor. The community was dominated by Cyanobacteria (37.59%) and Proteobacteria (34.96%) phyla followed by Firmicutes, Actinobacteriota and Bacteroidota. Within the Cyanobacteria phylum, 27% of relative abundance was attributed to *Desmonostoc* sp. and 5% to *Tetradesmus obliquus* green algae, which were reported to grow under harsh environmental conditions, utilizing recalcitrant substances and nutrients^59,67^. Degradation of organic substances, reflected by a COD decrease can be linked to the activity of bacteria, especially those attributed to the Proteobacteria, Firmicutes and Actinobacteriota phyla^68^. Moreover, numerous species of Proteobacteria are known to remove phosphorous and nitrogen from wastewater^69^. The removal of phosphates can also be linked to the appearance of bacteria belonging to the Chloroflexi phylum^70^. On the other hand, nitrogen transformation could have been influenced by Rhizobiales (14.63% relative abundance). Within this order, *Beijerinckiaceae*, *Rhizobiaceae* and *Xanthobacteraceae* were the dominant families, and these microbes could be responsible for nitrogen fixation and denitrification^71,72^. *Salinarimonas* sp. (4.29% of relative abundance) is known to grow in the pH range of 6–9, utilizes nitrates as a nitrogen source and is commonly found in substrates such as soil or piggery derived digestates^73,74^. Other denitrifying bacteria belonged to *Rhodanobacter* sp. within the Xanthomonadales order (10.45% relative abundance), and were previously reported to easily adapt to nitrogen-rich environments and low pH^75^. Furthermore, the appearance of *Sporosarcina* sp. (*Planococcaceae*) along with *Ferruginibacter* sp. (*Chitinophagaceae*) could enhance both nitrogen and COD removal as these bacteria are capable of simultaneous nitrification, denitrification and organic matter degradation^76^. Other bacteria detected belonged to the *Sphingomonadaceae* family with *Sandaracinobacter* sp. being the most abundant and *Comamonadaceae* dominated by *Comamonas* sp. A photoheterotrophic bacteria *Sandaracinobacter* sp. has been described as capable of degrading various organic compounds and growing at pH of 6–8. Species of these bacteria were previously found in soil, freshwater or sludge^77–79^. *Comamonas* sp., has been previously reported to reduce nitrites and accumulate polyphosphates^48,80^ (Fig. 4).

The majority of detected bacteria and cyanobacteria (i.e., *Desmonostoc* sp., *Salinarimonas* sp., *Rhodanobacter* sp., *Sporosarcina* sp, *Sandaracinobacter* sp., *Nitrosomonas* sp., *Nitrospira* sp., *Nitrosospira* sp.) inhabit environments within the pH range of 6–9 and are commonly found in digestates or agricultural substrates^59,67,73–75,77–79,81^. Bacteria native for the raw substrate did not directly influence the microalgal population as chlorophyll *a* concentration did not change with digestate feeding (Fig. 3a). It is believed that microalgae–bacteria consortia are more suitable for digestate treatment than axenic or non-axenic monocultures. During photosynthesis, microalgae produce oxygen used by aerobic bacteria for mineralization of organic compounds. In turn, native bacteria provide carbon dioxide necessary for microalgae. Higher pollutant treatment efficiency can be therefore achieved through such the symbiosis^12–14^.

## Conclusions

This study demonstrated that microalgae, bacteria and non-toxic cyanobacteria can be successfully used for the treatment of undiluted and unsterilized liquid digestate. For steady state period, nitrogen and phosphate removal rates reached 89.3 ± 0.5% and 72.8 ± 2.1% respectively. Furthermore, total COD was reduced by 78.9 ± 6.0% whereas soluble COD was lowered by 91.2 ± 1.6%. A significant pH change contributed to the rapid development of cyanobacteria, however nutrient removal efficiency remained at a relatively constant level. No MC-LR and MC-RR microcystin were detected. The investigation also proved that unsterilized, non-diluted liquid digestate can be successfully treated by a mixed consortium of microalgae, cyanobacteria and bacteria, and the growth of the latter two groups can enhance the treatment efficiency, especially the removal of nutrients. Further research is needed to find symbiotic relationships between microalgae, cyanobacteria and bacteria as well as mechanisms of contaminant removal at a proteolytic and metabolomic levels.

## Data Availability

The sequences obtained during the current study were submitted to the National Center for Biotechnology Information (NCBI) under accession no. OQ792152 and PRJNA922144 (BioSampleAcc.SAMN32643820).
